# Experiences of Structured Elicitation for Model-Based Cost-Effectiveness Analyses

**DOI:** 10.1016/j.jval.2018.01.019

**Published:** 2018-06

**Authors:** Marta O. Soares, Linda Sharples, Alec Morton, Karl Claxton, Laura Bojke

**Affiliations:** 1 Centre for Health Economics, University of York, York, UK; 2 Medical Statistics Department, London School of Hygiene and Tropical Medicine, London, UK; 3 Management Science, University of Strathclyde, Glasgow, UK; 4 Department of Economics, University of York, York, UK

**Keywords:** Bayesian, cost effectiveness, decision modeling, elicitation, expert judgment, subjective

## Abstract

**Background:**

Empirical evidence supporting the cost-effectiveness estimates of particular health care technologies may be limited, or it may even be missing entirely. In these situations, additional information, often in the form of expert judgments, is needed to reach a decision. There are formal methods to quantify experts’ beliefs, termed as structured expert elicitation (SEE), but only limited research is available in support of methodological choices. Perhaps as a consequence, the use of SEE in the context of cost-effectiveness modelling is limited.

**Objectives:**

This article reviews applications of SEE in cost-effectiveness modelling with the aim of summarizing the basis for methodological choices made in each application and recording the difficulties and challenges reported by the authors in the design, conduct, and analyses.

**Methods:**

The methods used in each application were extracted along with the criteria used to support methodological and practical choices and any issues or challenges discussed in the text. Issues and challenges were extracted using an open field, and then categorised and grouped for reporting.

**Results:**

The review demonstrates considerable heterogeneity in methods used, and authors acknowledge great methodological uncertainty in justifying their choices. Specificities of the context area emerging as potentially important in determining further methodological research in elicitation are between- expert variation and its interpretation, the fact that substantive experts in the area may not be trained in quantitative subjects, that judgments are often needed on various parameter types, the need for some form of assessment of validity, and the need for more integration with behavioural research to devise relevant debiasing strategies.

**Conclusions:**

This review of experiences of SEE highlights a number of specificities/constraints that can shape the development of guidance and target future research efforts in this area.

## Introduction

Reimbursement decisions are often supported by model-based economic evaluation (MBEE) [1]. Uncertainty in the evidence used to populate these models can result in uncertain cost-effectiveness estimates [2]. There may be circumstances in which empirical data are limited (e.g., a cancer product licensed on the basis of progression-free survival, with limited evidence on survival impacts) or are missing entirely (e.g., when assessing the value of a future clinical trial for a medical technology). In these situations, additional information, often in the form of expert judgments, reported as a distribution, is needed to reach a decision. To improve the accountability of the decision-making process, the procedure used to derive these judgments should be transparent, with any uncertainty in individual judgments characterized, in addition to between-expert variation [3].

Formal methods to quantify experts’ beliefs exist and are termed as structured expert elicitation (SEE) [3], [4]. Elicitation has been used in various disciplines including weather forecasting [5] and food and safety risk assessments [6]. Nevertheless, the existing methodological research on elicitation, both generic and discipline-specific, is inconsistent and noncommittal [7]. Methodological uncertainties may be one of the main reasons for the limited use of formal SEE in the context of MBEE. A review of applications in this area, published in 2013 [8], identified only a small number (14) of studies reporting the use of SEE. This review did not seek to determine the reasons for heterogeneity of approach, nor did it look at the challenges faced when conducting SEE to support MBEE and inform directions for future research.

In pursuit of further clarity, this article updates the aforementioned review [8], but instead of reporting the way elicitation is being used in practice, it focuses on summarizing the basis for methodological choices made in each application (design, conduct, and analysis) and the difficulties and challenges reported by the authors. In the Methods section, the methods for identifying the literature are described and an overview of the contexts in which SEE was used across studies is made. The sections that follow discuss choices, challenges, and issues relating to the design of SEE; conduct of SEE; and analyses of SEE. In detailing these elements it is necessary to first describe the applications (see the Summary of Applied Studies section and Table 1, Table 2, Table 3), and that is where the similarities exist between this review and the 2013 [8] review, and also where they end. The last section sets out specific challenges posed by SEE in MBEE to inform the direction of future research.

**Table 1:** Summary of applications

**Study**	**Type of strategy under investigation**	**Was the aim to inform an early assessment (i.e., R&D) rather than reimbursement?**	**Type of parameter(s) elicited**
Garthwaite et al. [14]	Treatment	No	Event probabilities, time to event, dependency
Leal et al. [10]	Diagnostic/screening	No	Event probabilities, relative effectiveness, diagnostic accuracy
Girling et al. [15]	Treatment	Yes	Event probabilities, time to event
Stevenson et al. [16]	Prevents transmission	No	Event probabilities, time to event, relative effectiveness
Meads et al. [12]	Diagnostic/screening	Yes	Event probabilities, diagnostic accuracy, minimum important clinical difference
McKenna et al. [19]	Treatment	No	Event probabilities
Haakma et al. [13]	Diagnostic/screening	Yes	Diagnostic accuracy
Stevenson et al. [17]	Treatment	No	Event probabilities, relative effectiveness
Speight et al. [25]	Diagnostic/screening	No	Event probabilities
Sperber et al. [22]	Treatment	No	Event probabilities, relative effectiveness
Brodtkorb [26]	Several exercises conducted but insufficient detail reported on each		
Colbourn et al. [28]	Diagnostic/screening	No	Event probabilities, relative effectiveness
Soares et al. [9]	Treatment	No	Event probabilities, relative effectiveness
Bojke et al. [18]	Treatment	No	Relative effectiveness, dependency
Cao et al. [11]	Diagnostic/screening	Yes	Relative effectiveness
Fischer et al. [23]	Treatment	No	Counts, time to event
Poncet et al. [27]	Diagnostic/screening	No	Event probabilities
Grigore et al. [24]	Treatment	No	Event probabilities
Wilson et al. [20]	Treatment	No	Event probabilities, relative effectiveness
Meeyai et al. [21]	Vaccine	No	Event probabilities
Grimm et al. [35]	Diagnostic/screening	No	Diffusion†

R&D, research and development.

† Rate of implementation in clinical practice over time.

**Table 2:** Experts, method of elicitation, and method of aggregation

**Study**	**Experts and recruitment**			**Approach and method of elicitation**		**Aggregation**		
	**Type of experts**	**Recruitment**	**No. of experts**	**VIM or FIM**	**Method (summaries elicited)**	**Aggregation approach**	**Weights used in main exercise?**	**Nature of weights**
Garthwaite et al. [14]	NR	NR	4	VIM	Median and quartiles	Mathematical	No	–
Leal et al. [10]	Clinicians	Purposive	6	FIM	Four complementary intervals	Mathematical	No	–
Girling et al. [15]	Clinicians	Purposive	5	VIM*	NR	Consensus	–	–
Stevenson et al. [16]	NR	NR	NR	NR	NR	NR	NR	NR
Meads et al. [12]	Clinicians	Purposive	21	FIM	Chips and bins†	Mathematical	No	–
McKenna et al. [19]	NR	NR	5	FIM	Chips and bins	Mathematical	No	–
Haakma et al. [13]	Clinicians	Purposive	14	VIM	Mode and 95% CI	Mathematical	Yes	Objective weights
Stevenson et al. [17]	Clinicians	Purposive	3	VIM	Median and quartiles	Consensus	–	–
Speight et al. [25]	Clinicians	NR	9	FIM	Chips and bins†	NR	NR	
Sperber et al. [22]	Clinicians	NR	NR	VIM	Median and quartiles	Mathematical	Yes, but no details provided	Performance-based
Colbourn et al. [28]	NR	NR	4	VIM	Mean and 95% CI	Mathematical	NR	NR
Soares et al. [9]	Clinicians	NR	23	FIM	Chips and bins	Mathematical	No, but explored in a pilot	Performance-based weights explored
Bojke et al. [18]	Clinicians	Purposive	5	FIM	Chips and bins	Mathematical	Yes	Performance-based weights
Cao et al. [11]	Clinicians	NR	2	FIM	Mode and one percentile	Mathematical	No	–
Fischer et al. [23]	Clinicians	Purposive	19	VIM	Median and 80% CI	Mathematical	No, but explored	Performance-based weights explored
Poncet et al. [27]	Clinicians	NR	13	FIM	Chips and bins	Mathematical	No	–
Grigore et al. [24]	Clinicians	Purposive	7	FIM	Chips and bins + four complementary intervals	Mathematical	Yes, alongside equal weighting	Performance-based weights explored
Wilson et al. [20]	Clinicians + policy strategist	NR	6	NR	NR	NR	NR	NR
Meeyai et al. [21]	Clinicians + epidemiologists	NR	10	VIM	Mode and quartiles	Consensus	–	–
Grimm et al. [35]	NR	NR	3	NR	NR	Mathematical	NR	NR

CI, confidence interval; FIM, fixed interval method; NR, not reported; VIM, variable interval method.

*Unclear, but description of results suggests that VIM has been used.

† Includes studies that list the following methods: chips and bins, frequency chart, and histogram method.

**Table 3:** Summary of applications (conduct and analyses)

**Study**	**Conduct**					**Analyses**		
	**Mode of administration**	**Opportunities for revision**	**Format/software**	**Training**	**Piloting**	**Pooling**	**Fitting**	**Pooled distribution used directly within the decision model?**
Garthwaite et al. [14]	Individual face-to-face and remote (telephone) interviews	Unclear	Interview and specialized software	NR	No	No pooling	Independently elicited quantities: NR; dependency elicitation: yes, generalized linear model	No, each expert’s distributions used directly
Leal et al. [10]	Remote (email) and individual face-to-face interviews	Yes	Excel-based	NR	Yes	Linear pooling	Yes, maximum likelihood	Yes
Girling et al. [15]	Group face-to-face interviews	Yes	NA	NR	NR	–	Yes, method NR	Yes
Stevenson et al. [16]	NR	NR	NR	NR	NR	NR	NR	Yes
Meads et al. [12]	Group face-to-face and individual face-to-face interviews	NR	Paper	Yes	NR	Linear pooling	NR	No, Bayesian updating with existing evidence
McKenna et al. [19]	NR	NR	Excel-based	Yes	Yes	Linear pooling	Yes, method NR	Yes
Haakma et al. [13]	Individual face-to-face interviews	Yes	Excel-based	NR	Yes	Linear pooling	Yes, Project Evaluation and Review Technique software	Yes
Stevenson et al. [17]	Group face-to-face interviews	Yes	NR	NR	NR	NA	Yes, least squares	NR
Speight et al. [25]	NR	NR	Paper	NR	NR	NR	Yes, method NR	Yes
Sperber et al. [22]	Remote (telephone) interviews	Yes	Excel-based	NR	Yes	Linear pooling	Yes, least squares (EasyFit software)	NR
Colbourn et al. [28]	NR	NR	NR	NR	NR	Predictive distribution from random-effects meta-analysis	Yes, method NR	Yes
Soares et al. [9]	Group face-to-face interviews	Yes	Excel-based	Yes	Yes	Linear pooling	Yes, method of moments	No, Bayesian updating with existing evidence
Bojke et al. [18]	Individual face-to-face interviews	Yes	Excel-based	Yes	NR	Linear pooling and random-effects meta-analysis	Yes, method of moments	Yes
Cao et al. [11]	NR	NR	NR	NR	NR	Linear pooling	Yes (BetaBuster software)	Yes
Fischer et al. [23]	Group face-to-face, individual face-to-face, and remote (telephone) interviews	NR	Paper	Yes	Yes	Linear pooling	No, empirical distribution used	NR
Poncet et al. [27]	NR	NR	NR	NR	NR	Linear pooling	NR	Yes
Grigore et al. [24]	Individual face-to-face interviews	Yes	Excel-based	Yes	Yes	Linear pooling	Yes, method NR	Yes
Wilson et al. [20]	NR	NR	SHELF	NR	NR	NR	NR	Yes
Meeyai et al. [21]	NR	NR	SHELF	NR	NR	NR	NR	No, Bayesian updating with existing evidence
Grimm et al. [35]	NR	NR	NR	NR	NR	Linear pooling	Yes, least squares	Yes

NA, not applicable; NR, not reported; SHELF, Sheffield Elicitation Framework.

## Methods

To identify applications of SEE, the 2013 review [8] was updated (identifying studies up to April 11, 2017). Further details on the methods of the search are given in the Appendix in Supplemental Materials found at doi:10.1016/j.jval.2018.01.019, but, in brief, studies were identified via Ovid SP MEDLINE and, similarly in the 2013 review [8], were included only if they contained an SEE to elicit uncertain parameters (in the form of a distribution) to inform MBEE. Studies conducting preference elicitation(e.g., to generate utility estimates for health states) were not included.

The methods used in each application were extracted (the extraction form is reproduced in Table 1, Table 2, Table 3, which also present results) along with the criteria used to support methodological and practical choices and any issues or challenges discussed in the text. Issues and challenges were extracted using an open field and then categorized and grouped for reporting.

## Results

### Summary of Applied Studies

In total, 21 studies were included. Table 1 and the Appendix in Supplemental Materials provide summary information on each study and highlight that elicitation has been used mainly when data on a particular parameter are limited or absent. Four of the 21 applications were applied in an early modeling context, where there may not be direct clinical experience with the technology of interest, and 8 evaluated a diagnostic or screening strategy.

Table 2 presents the method of recruiting experts, methods of elicitation, and methods of aggregation in each of the applied studies. Table 3 presents how the SEE was conducted, including mode of administration and use of any software, and also any analyses that were performed. Each element of the applied studies is considered, and choices, challenges, and issues discussed in the following sections.

### Aspects Related to the Design of the SEE

Considerations on the design of the SEE were grouped according to specification of the quantities to elicit, selection of experts, elicitation method, and type of aggregation and weighting of experts’ judgments.

#### Specification of quantities to elicit

In all applications, experts’ beliefs were sought for only a few parameters of a decision model, often not elicited directly but calculated from one or more alternative elicited quantities. For example, a time-constant transition probability could be indirectly elicited by asking experts for the mean time at which an event is observed or, alternatively, the proportion of patients who have an event within a particular time period. In the applications, the choice of which quantities to elicit was based on a number of criteria. The first was appropriateness for experts. Parameters in decision models can be complex and may not be directly observable by experts; to account for this, some of the studies expressed, for example, relative effectiveness parameters as probabilities [9], [10], [11], [12] or sensitivities and specificities into probabilities of the true disease status of the patients conditional on the test results [12]. It may also be more appropriate for different experts to elicit different quantities (e.g., in one application [10], geneticists elicited accuracy of a genetic test, and cardiologists elicited parameters related to disease progression) or, in the presence of heterogeneity, for a particular quantity to be elicited separately for population subgroups [12], [13].

The second criterion related to statistical concerns. The quantities elicited should be fit-for-purpose not only in informing decision models (e.g., reflecting time dependency), but also in allowing elicited evidence to be combined with any existing empirical evidence [9]. Statistical coherence between quantities elicited should also be ensured [9], [14]. For example, when a number of mutually exclusive outcomes are of relevance, eliciting their probabilities independently (with uncertainty) cannot guarantee that they sum to 1, but re-expressing parameters as conditional binomial variables does ensure this [9], [14]. In addition, dependencies may exist between the quantities elicited (e.g., correlation between relative effectiveness parameters for alternative interventions), between quantities elicited and known covariates, or between a priori independent quantities that are elicited from the same expert (e.g., some experts may be prone to eliciting higher values across the board than others). Of the seven studies that raised the issue of dependency [9], [11], [14], [15], [16], [17], [18], three did not deal with it at all [15], [16], [17]; two studies re-expressed target parameters as conditionally independent [9], [11] and the remaining two studies explicitly elicited dependency [14], [18]. In the latter, rather than a correlation parameter being elicited directly, relationships between parameters were captured by asking experts to express how their judgments would change if values for other quantities were known. Methods to elicit dependence directly were, however, generally thought to be complex [9].

The final criterion was burden to experts. Burden can be reduced by limiting the number of target parameters to elicit, eliciting homogeneous quantities throughout the exercise (e.g., all probability parameters) [9], using filter questions (e.g., “Do you think X differs from Y?”) [9], [19], and not eliciting dependency or eliciting it only for the covariates identified by the experts as relevant [14].

#### Selection of experts

All applications recruited health care professionals (but not exclusively [20], [21]) on the basis of the following criteria: recognition by peers [10], specialist knowledge or clinical experience [9], [10], [13], [18], [19], [22], [23], based in the relevant jurisdiction [9], [10], [18], [19], research experience [10], [22], [23], and lack of involvement in product development [13]. In early technology assessment, applications have also looked for other factors such as interaction with colleagues, seen as indicative of the adaptive skills required in this context.

A number of authors [9], [14], [24] recognized that health care professionals are unlikely to have knowledge of elicitation and may have only sparse quantitative skills. This has been judged by the authors to compromise normative skills, defined as the ability to accurately express judgments in a particular quantitative format, such as probabilities. This has driven the choices made in designing and conducting the SEE, such as training needs, method of elicitation, and definition of the quantities to elicit [9].

Many of the applications have included a varied sample of experts by recruiting them from a range of relevant specialties [10], [12], [20], clinical settings [9], [10], [20], and geographical areas/countries [10], [23] to capture heterogeneity in beliefs (reflecting underlying heterogeneity in patient populations) and avoid dependency between experts [10].

Across the applications, sampling was purposeful: typically, experts recruited were either collaborators in the research or identified by recommendation from clinical colleagues [10], [14], [18], by contacting professional associations [24], or at specialist conferences [15], [23]. Sample sizes ranged from 2 [11] to 23 [9] (Table 2), generally targeting a small but “varied” sample [10]. One author [22], however, argued that restricting the pool of experts may amplify biases arising, for example, from shared exposure to unrepresentative clinical experience. Many applications mention constraints to sampling because of resources available to fund the SEE [22], limited number of relevant experts [10], or geographic distance [18], [22].

#### Elicitation method

An important requirement for MBEE is the need to elicit uncertainty of experts’ judgments in the form of a distribution. This implies that a number of summaries need to be elicited for each quantity to define the shape of a distribution. To do this, applications have typically used one of two approaches: fixed interval method (FIM) [9], [10], [11], [12], [18], [19], [24], [25], [26], [27] or variable interval method (VIM) [13], [14], [17], [21], [22], [23], [28] (Table 2). In an FIM, experts are provided with ranges of values and asked to assess the probability that the quantity lies in each. In a VIM, experts are asked to specify values of the quantity of interest for predefined percentiles of the distribution. Although one application [10] chose FIM because the literature suggested that it returns higher variance, it was more common for authors to consider both approaches. Choices were justified on the basis of pilot exercises designed for the purpose (see later), generic methods research, previous use in MBEE, and claims of lower burden or intuitiveness for experts.

Applications using VIM elicit either quartiles of the distribution [14], [17], [21], [22] or credible intervals [13], [23], [28], and in general ask for a very limited number of summaries. Studies using FIM often choose the “chips and bins” method (histogram technique or probability grid) [9], [18], [19], [25], [26], [27]. This method defines a larger number of intervals (typically up to 20) and asks the expert to distribute a fixed number of chips across these intervals. The more chips placed in a particular interval, the stronger the belief that the true value of the quantity of interest lies in that interval. Despite many of the studies arguing for the intuitiveness of the chips and bins method, a pilot study [13] found that two of the three experts included preferred eliciting 95% probability intervals. Other FIMs, which divide the plausible range of values into four or six complementary or overlapping intervals and ask for quantitative expression of strength of belief for each, have also been used [10]. Pilot testing among these found that six complementary intervals resulted in very narrow ranges and that overlapping intervals were confusing to experts [10]. A separate study [24] comparing the chips and bins method with the four complementary intervals method found that the latter required more careful consideration and, because of that, experts perceived it to be more (face-) valid. Also, the resulting pooled distributions were wider. Another FIM application [11] asked experts for a central estimate and elicited for a single interval. This study was noteworthy because it took a more frequentist approach by presenting a hypothetical scenario in which 100 different experiments were conducted and asked experts how many times in those experiments would they expect the observed value to be larger than a particular value.

#### Consensus versus mathematical aggregation, weighting of experts

Fourteen studies [9], [10], [11], [12], [13], [14], [18], [19], [22], [23], [24], [27], [28], [35] elicited individually from experts and aggregated mathematically, three aimed to achieve consensus among experts [15], [17], [21], and three others did not explicitly report the method of aggregation used [16], [20], [25] (Table 2).

None of the three studies using consensus were explicit about the reasons for choosing consensus or the process of achieving it. Therefore, the following focuses on those using a mathematical approach.

Authors justify the choice of mathematical aggregation on the basis of the desirability to reflect variation within and between experts [12], because consensus is known to lead to overconfident results (i.e., narrow distributions) [10] and because it raises practical difficulties of convening experts and providing experienced facilitation. One pilot study [9] additionally showed that consensus produced incoherent probability statements (the median time to healing was greater than the time taken for 70% of patients to heal).

When adopting a mathematical approach there needs to be some consideration on whether to differentially weight the responses of individual experts. Most of the applications reviewed claim insufficient justification for generating differential weights [9], [10] and lack of clarity on how to appropriately generate the weights [9], [13], [26] and hence apply equal weighting. Five studies, however, explored unequal weighting, either on the basis of responses to “seed” questions [9], [18], [23], [24] (performance-based weighting) or using the clinical background of experts [13] (objective weighting). Performance-based weighting (commonly called calibration) typically asks experts to respond to one or more seed questions known to the analyst (with certainty) but not the expert. Elicited responses are compared with their known values to generate the weights, with the most commonly used method, the classical method [29], considering both accuracy and informativeness.

Applied studies question the usefulness of calibration and request further methodological research. Uncertainties relate to the number and definition of appropriate seed questions for particular target questions. For example, one study [9] piloted four alternative seed questions and found that, when used separately, these generated divergent weights. In another application, responses to eight seed questions generated 0 weights to 17 of the 19 substantive experts—authors expressed discomfort in discarding so much of the information. A third study [18] questioned the relevance of seed questions known with certainty and instead used seeds that were known with uncertainty. Weights were generated on the basis of the overlap of the elicited and true distributions.

### Experiences with the Conduct of the Exercise

No studies reported major challenges in the conduct of the SEE, despite the complexity of the task.

Consensus exercises were typically face-to-face, in a group; mathematical exercises adopted a mix of formats, ranging from individual interviews to remote completion via email (Table 3). Convening a group facilitated training and a common understanding of the problem [12], but some studies [10], [12], [14], [26] departed from this format because of time constraints, geographical limitations, and constraints related to the availability of experts. One example [9], using a mathematical approach, elicited 18 uncertain parameters in 2 hours after a 2-hour training session.

Administration was via bespoke tools using Excel [9], [10], [18], [19], [24], [26], paper questionnaires, a generic elicitation package (the Sheffield Elicitation Framework) [20], [21], and a software package for the elicitation of dependency (Prior Elicitation Graphical Software) [14] (Table 3). Other studies did not specify the mode of administration. The most common tool, bespoke Excel applications, had several perceived advantages, including tailoring the presentation to avoid inconsistencies and conditioning questions on previous responses [9].

Some exercises were explicit about piloting the tool to ensure clear wording of the questions [9], [13], [19], [22], and most offered opportunities for revision and/or graphical feedback (Table 3).

Five applications were explicit about training of experts [9], [12], [13], [18], [24] covering overview of the project and of the role of elicitation [9], [12], [13], [18], [24], quantities required and definitions [9], [12], [18], [24], explanation and expression of uncertainty [9], [13], consideration of potential biases [9], [18], [24], use of the elicitation instrument [9], and delivery of practice exercises [9], [12], [18], [24]. Studies that implemented elicitation remotely generally included some form of instructions, although none reported these in detail [10].

### Experiences with the Analyses and Interpretation of Elicited Evidence

Considerations on the experiences of analyses and interpretation of elicited evidence were grouped according to validity assessment, syntheses of multiple beliefs for mathematical aggregation, deriving smooth prior distributions, and further use of elicited evidence in decision modeling.

#### Considerations on validity

Aspects related to validity in applied studies were missingness, validity checks, and self-reported face validity. Reporting of missingness was poor: no applications provided recruitment rates, only a few provided the number of recruited experts who did not turn up or did not return the elicitation form [10], [11], [12], [14], [24], and none was explicit about missing responses to individual questions. No studies dealt with missing responses, either formally or informally.

Three types of validity checks have been implemented. One study [22] contrasted qualitative and quantitative responses (internal validity) and found a small number of inconsistent responses; for example, the statement “I don’t know, this isn’t my area of research” was accompanied by extremely certain probability estimates. A second type of validity check compared the elicited beliefs of multiple experts [9], [11], [14], [23], [25], [26]. Although some authors [12], [14] valued good agreement, others [9], [10] accepted variation between individuals (on the basis that individual beliefs are being requested). Finally, when external evidence was available, this was compared with elicited beliefs (external validity) [9], [12], [14], [26]. Authors sought agreement, but when differences arose they cautiously justified them on the basis of population differences [12].

Some applications requested feedback from experts on the ease of completion of the SEE [9], [10], [24], [26], the basis for experts’ answers (to reveal the sources of evidence considered by the experts and their level of knowledge [10]), or self-reported face validity [9], [10], [12], [24].

#### Syntheses of multiple beliefs elicited in mathematical aggregation

Of the 14 studies that used a mathematical approach to aggregation, 1 did not generate a group estimate and instead used the responses of each expert individually [14]. Nine linearly pooled, by averaging individual distributions (with or without weighting; see earlier). Authors justify this choice on the basis of the lack of published evidence that more complex methods outperform linear pooling [10]. Two other studies used the predictive distribution from a random-effects meta-analysis of individual elicited distributions [18], [28], a method arising from statistical methodology rather than the wider elicitation literature. Given the random-effects model results in a combined distribution that can be more precise than any of the individual distributions, this pooling method has been deemed inappropriate for use in MBEE [18].

Generally, inputs to decision models were pooled across experts, except in one study that ran the model with each of the individual elicited distributions and linearly pooled resulting outputs [11].

#### Deriving smooth prior distribution functions

Some applications were not explicit about how prior distributions were derived from elicited summaries. Those that were explicit used parametric distributions (Table 3), with the choice of distribution either not justified or based on general MBEE literature on distribution choice for probabilistic sensitivity analyses [10]. To fit the distribution (i.e., evaluate the parameters of the distribution that best fit the empirical distributions elicited from experts), some applications cited software [11], [13] and others cited the fitting method (e.g., maximum likelihood fitting [10], least squares [17], and method of moment [9], [18]). Goodness of fit was evaluated either in discussion with the experts [17] or graphically by superimposing the fitted probability density function on the histogram [10]. Bojke et al. [18] acknowledged that although the fit of prespecified parametric distributions was not always ideal in their example, methods that allow fitting of nonparametric distributions are more complex and can complicate further analyses, particularly when Bayesian updating is further required, for example, in value of information calculations.

#### Further use of elicited evidence in decision modeling

Elicited evidence has been seen as a way of characterizing uncertainty for model parameters or assumptions to inform the decision to acquire further evidence [9]. In some applications, elicited evidence was used directly as input to the cost-effectiveness model [10], [12], [14], [15], [20], [24]. When external evidence existed on elicited parameters, some authors presented both sources separately using scenarios [12], [17], whereas others combined them using Bayesian updating [9], [16], [21]. The latter is consistent only under the assumption that the experts did not consider existing evidence when formulating their judgments [9]. Three authors [11], [14], [19] explored use of individual experts’ beliefs and found that results and associated allocation decisions varied between experts.

### Considerations on Bias

When seeking to gather experts’ opinions, it is important to consider their potential biases, specifically motivational or cognitive [30]. Motivational biases relate to conscious or subconscious distortions of judgments because of self-interest. Cognitive biases are associated with the use of heuristics: cognitive shortcuts that individuals use when asked for complex judgments. When such mental processes are faulty, these may lead to biased judgments.

The potential for bias in expert opinion was recognized in some SEEs [9], [22] with reported attempts to minimize bias in the design [26]. Two applications made explicit efforts to avoid recruiting experts that may have motivational biases [13], [20]. Two studies provided information on cognitive biases in the training session [9], [24].

## Conclusions

In the published applied studies, authors generally recognized great potential for using elicitation in MBEE, particularly when evidence was absent (including the early modeling context [13]).

Our critical review demonstrates that reporting is poor (as also identified elsewhere [31]), and there is a lack of consensus on methodology. Given the direct link to healthcare policy decisions, it is important that methodological guidance specific to health technology assessment (HTA) is generated, with consideration of the constraints inherent to the processes of policy decisions (such as timelines, budget, and availability of experts). A number of principles from the elicitation literature are expected to generalize to the MBEE setting, such as the need for piloting and training; nevertheless, for many other areas of SEE, it is not clear that methods used in other disciplines translate to HTA. Our review highlights a number of specificities/constraints that can shape the development of guidance and target future research efforts in this area, summarized as follows.

First, there exists important between-expert variation. In other disciplines, variation is generally linked to different levels of bias and hence regarded as undesirable, warranting the use of strategies to reduce or discourage variation, such as consensus methods. Most applications in MBEE, however, expect wide variation in the beliefs of multiple experts because of genuine heterogeneity in the populations experts draw upon. Further research efforts should examine the origins of variation and consider how to appropriately reflect it.

Second, substantive experts in HTA are health professionals who may not be trained in quantitative subjects, unlike other areas of science in which elicitation is used such as engineering or meteorology. Further research on SEE should consider the appropriateness of alternative methods of elicitation (e.g., chips and bins method or bisection method) for the potentially less normative experts, or how to facilitate the elicitation of complex parameters, including dependency. Furthermore, elicitation may have an important role in early modeling in which experts’ beliefs are required on new technologies. In this case, adaptive skills are required to allow experts’ substantive expertise (in the disease area and/or other health care strategies) to be appropriately used. Further research should focus on how to promote the use of adaptive skills or how to determine better performing individuals in this context.

Cost-effectiveness modeling typically requires judgments on a relatively large number of parameter types (e.g., probabilities, relative treatment effects, costs, and health-related quality-of-life scores), and the design and conduct of an SEE may well be influenced by what quantities are required. The applications reviewed here elicit a range of different quantities to inform the same parameter; they, however, do not draw on evidence or past experiences specific to that quantity. Design of future applications could be aided by a compilation of possible quantities that can be reasonably used to elicit particular parameters types, accompanied by guidance on how to ensure that the multiple quantities elicited in a particular application can be appropriately used within a decision model.

Perhaps given the direct link to decision making, most applied examples seek for assurance on the validity of the particular exercise. It is, however, not clear how such an assessment should proceed. Examples have used self-reported face-validity assessments, sensitivity analyses, and performance weighting (calibration). Particularly for performance weighting, despite a growing (generic) literature discussing the validity of this approach (see, e.g., Refs. [32], [33], [34]), the applied literature struggles with supporting the methodological choices that need to be made. Although some means of correcting for poor performance are welcomed, in the applied literature, concerns have been expressed that this should not repress expressions of heterogeneity. If SEE is to be used more systematically in MBEE, further guidance is needed on how to demonstrate validity.

Finally, although it is generally agreed that SEE should be designed and conducted in a way that minimizes the use of heuristics and other sources of bias, there is little integration in the applied literature of the findings from behavioral research. A recent review placing special emphasis on debiasing techniques [30] is a helpful resource to be reflected on in future research.

It is worth noting that our review includes only published examples, despite SEE being conducted more widely for MBEE. Moreover, the review is based on analytical reading of the published articles, and is hence subject to a certain amount of interpretation. Further research in understanding the landscape of SEE for MBEE could include structured discussions among individuals with experience in the area to explore challenges in past exercises and identify those foreseen in future applications.

Source of financial support: Work contributing to this article was conducted as part of a Medical Research Council grant “HEE: Developing a reference protocol for expert elicitation in health care decision making” (grant no. MR/N028511/1). L. Bojke was also supported in the preparation/submission of this article by the Health Economics and Outcome Measurement Theme of the National Institute for Health Research Collaboration for Leadership in Applied Health Research and Care Yorkshire and Humber (grant no. IS-CLA-0113-10020). The views and opinions expressed are those of the authors, and not necessarily those of the Medical Research Council, the UK National Health Service, the National Institute for Health Research, or the Department of Health.

## Footnotes

Supplemental data associated with this article can be found in the online version at 10.1016/j.jval.2018.01.019.
