# Supplementary material for: Experiences of Structured Elicitation for Model-Based Cost-Effectiveness Analyses
Source: Value Health. 2018 Jun;21(6):715–23. doi: 10.1016/j.jval.2018.01.019 (PMC6021555; doi:10.1016/j.jval.2018.01.019)
Supplement: Supplementary file 1 — Supplemental Materials [file mmc1.docx]

**Supplementary material**

| **Study** | **Aim of the study and of the elicitation** | **Elicitation exercise informing a standard decision model?*** |
| --- | --- | --- |
| Garthwaite 2008^14^ | To evaluate the cost-effectiveness of alternative models for bowel cancer service provision in England. For some quantities information was only available in the background knowledge and experience of experts. The study describes the elicitation process. | Yes |
| Leal 2007^10^ | Economic evaluation aimed at assessing the long-term costs and effects of genetic and non-genetic diagnostic approaches in Hypertrophic Cardiomyopathy for those at risk of sudden cardiac death. There was limited evidence on several model parameters. In the absence of data, distributions were elicited from experts. | Yes |
| Girling 2007^15^ | The aim of this study was to establish the outlook for the second generation of left-ventricular assist device (LVAD) implantation as a therapy in end-stage heart failure, specifically in terms of survival benefit and implications to cost-effectiveness and Bayesian value of information analyses. Prior distributions were elicited probabilistically from a group of leading experts to inform parameters which there was no direct empirical evidence. | Yes |
| Stevenson 2009^16^ | This paper describes the modelling and analysis of surgical instrument replacement and management policies for certain operations that may present a risk to human patients for transmitting and contracting variant Creutzfeldt–Jakob disease. Important parameters to the model are poorly known due to lack of data; a panel of experts were therefore convened to describe the uncertainties that surround those parameters using probability elicitation techniques. | Dynamic model |
| Meads 2013^12^ | This project evaluated the cost-effectiveness of adding positron emission tomography/computerised tomography (PET-CT) as an adjunct to standard practice to diagnose recurrent cervical cancer. A subjective elicitation exercise was developed that aimed at informing the economic model in the absence of information found in the literature. | Yes |
| McKenna 2009^19^ | To determine the clinical- and cost-effectiveness of enhanced external counterpulsation (EECP) compared with usual care and placebo for refractory stable angina and heart failure, and to assess the potential value of future research on EECP. The elicitation of unknown parameters was conducted to evaluate the potential long-term cost-effectiveness of EECP. | Yes |
| Haakma 2014^13^ | Determine an early health economic model of photoacoustic mammography (PAM) imaging versus magnetic resonance imaging (MRI) for detecting breast cancer. The aim of the study was to elicit beliefs on diagnostic performance for the early stage of development diagnostic technology. | Yes |
| Stevenson 2009b^17^ | This study evaluates, in the context of England and Wales, whether it is cost-effective to conduct a randomized controlled trial (RCT) and what sample size may be optimal to estimate the efficacy of bisphosphonates in fracture prevention beyond 5 years. Two scenarios were run. The 1st uses long-term efficacy data from published literature, and the 2nd uses distributions elicited from clinical experts. | Yes |
| Speight 2006^25^ | To use a decision-analytic model to determine the incremental costs and outcomes of alternative oral cancer screening programmes conducted in a primary care environment. Expert opinion was used when no other data source was identified to inform model parameters. | Yes |
| Sperber 2006^22^ | Economic evaluation of a multicentre clinical trial in sleep apnea related to acute quadriplegia. The aim of the study was to develop an Excel tool for expert elicitation that can be used by remote completion, that will ultimately inform cost effectiveness modelling, | Yes |
| Brodtkorb 2010 | Performed several elicitation exercises, but detail on each is sparse |  |
| Colborn 2007^28^ | To determine the cost-effectiveness of prenatal strategies for preventing group B streptococci (GBS) and other serious bacterial infections in early infancy and to establish the expected value of further information. As no research data were available for the treatment effects of vaccination and of antibiotic treatment on stillbirths, estimates from experts were used. | Yes |
| Soares 2011^9^ | To determine the cost-effectiveness of negative pressure wound therapy and comparator dressings for the healing of severe pressure ulcers. The evidence base was limited and sparse but there was substantial practical experience of using the treatment and its comparators in clinical practice. Elicitation is used to capture this knowledge quantitatively. | Yes |
| Bojke 2010 ^18^ | These were used within a model developed to assess the cost-effectiveness of infliximab and etanercept for the treatment of active psoriatic arthritis (PsA), compared with palliative care. Elicitation can be used to characterize structural uncertainty within a decision analytic model. This allows the value of acquiring further evidence to resolve these uncertainties to be established. | Yes |
| Cao 2013^11^ | Analyses of the commercial headroom available to a novel point-of-care testing (POCT) device, which is defined as laboratory testing at or near the patient, in the disease management of patients with heart failure (HF). Probability elicitation has been used in early health economic modelling. | Commercial headroom analyses |
| Fischer 2013^23^ | The aim of this study was to use expert judgement elicitation to estimate currently unavailable key parameters for treatment models in severe haemophilia A. | Yes |
| Poncet 2015^27^ | To estimate the cost-effectiveness of electrocardiographic screening to detect Long QT in psychiatric inpatients. As the risk of Risk of Torsades-de-Pointes (an important outcome of a long QT) in patients with LQT is poorly characterized in the literature, it was derived from an experts’ elicitation | Yes |
| Grigore 2016^24^ | The elicitation exercise aimed to inform a published model-based cost-effectiveness analysis of degarelix versus triptorelin for the treatment of advanced hormone-dependent prostate cancer. This study had three objectives: (i) to obtain subjective probability distributions characterising parameter uncertainty in the context of a health technology assessment; (ii) to compare two elicitation methods by eliciting the same parameters in different ways; (iii) to collect subjective preferences of the experts for the different elicitation methods used. | Yes |
| Wilson, 2016^20^ | To estimate the cost-effectiveness of a 5-day course of IV thiamine, vs a 2- and 10-day course, in harmful or dependent drinkers presenting to emergency departments (EDs) with possible Wernicke’s encephalopathy (WE). Expert opinion via structured elicitation was used to derive some of the inputs required for the model. | Yes |
| Meeyai, 2015^21^ | Evaluate the cost-effectiveness of influenza vaccination policies in developing countries and used it to consider annual vaccination of school- and preschool-aged children with either trivalent inactivated influenza vaccine (TIV) or trivalent live-attenuated influenza vaccine (LAIV) in Thailand. This study adopted a Bayesian evidence-synthesis framework, and used prior probability distributions derived from literature review and elicitation of expert opinion. Elicited priors were used for parameters that either had not been reliably estimated or had been estimated only in locations where their values were thought likely to differ substantially from those in Thailand. | Dynamic model |
| Grimm 2017^35^ | To extend existing methods for assessing the value of research studies in terms of both reduction of uncertainty and improvement in implementation by considering diffusion based on expert beliefs with and without further research conditional on the strength of evidence. The framework of analyses developed is applied to a real case study of a preterm birth screening technology. | Value of implementation |
