# Supplementary material for: Experiences of Structured Elicitation for Model-Based Cost-Effectiveness Analyses
Source: Value Health. 2018 Jun;21(6):715–23. doi: 10.1016/j.jval.2018.01.019 (PMC6021555; doi:10.1016/j.jval.2018.01.019)
Supplement: Supplementary file 2 — Supplementary material [file mmc2.docx]

**Appendix**

Review methods: All of the studies included in the review by Grigore et al 2013^8^ were included in this review. Additionally, we updated this earlier review. The search strategy (described below) was taken from the Grigore search, and was applied in Ovid SP MEDLINE, with a date restriction spanning 2013 to the 11th April 2017. References and quotation checking were not conducted for this update.

Search Strategy:

1. elicit*

2. subjective ADJ1 probabilit*

3. bayes* AND prior$

4. probability ADJ1 distribution$

5. value ADJ3 information

6. probabilistic sensitivity analysis

7. 1 OR 2 OR 3 OR 4 OR 5 OR6

8. HTA

9. technology ADJ1 assessment$

10. cost-effectiveness

11. model*

12. comparative ADJ1 effectiveness

13. 8 OR 9 OR 10 OR 11 OR 12

14. expert$ ADJ1 elicitation

15. expert$ ADJ1 opinion

16. expert$ ADJ1 knowledge

17. expert$ ADJ1 judgement

18. expert$ ADJ1 belief$

19. expert$ ADJ1 panel$

20. advisor$ ADJ1 board$

21. 14 OR 15 OR 16 OR 17 OR 18 OR 19 OR 20

22. 7 AND 13 AND 21

Inclusion and Exclusion Criteria: The inclusion and exclusion criteria used in the search were drawn from the earlier review^8^. In summary, after the removal of duplicates, screening was undertaken by one reviewer (MS). Articles were included for full text screening if they described the use of expert opinion with the purpose of informing a cost-effectiveness model . Articles screened in full text were excluded if they aimed to elicit only point estimates (not probability distributions), or did not describe any aspect of the elicitation methods used. Where several papers described aspects of the same elicitation exercise, only the reference containing the most complete account of the elicitation was included.

Extraction: A data extraction tool was developed specifically for this task and aimed to collect information on the methodological choices made in each of the exercise – as reproduced in Tables 1 to 3. For each of these fields, information was extracted on the justification for the choices (using an open field). An additional open field was used to extract challenges with the design, conduct and analyses of the SEE, reported throughout the text and in the discussion. These were later categorised and grouped for reporting.

PRISMA Flow diagram for the search update

Studies included in qualitative synthesis
(n = 7 )

Records identified through database searching
(n = 81 )

Full-text articles excluded
(n = 8)

Full-text articles assessed for eligibility
(n = 15 )

Records excluded
(n = 60)

Records screened
(n = 75 )

Records after duplicates removed
(n = 75 )

## Identification

## Eligibility

## Included

## Screening
